# Cortical Processing Related to Intensity of a Modulated Noise Stimulus—a Functional Near-Infrared Study

**DOI:** 10.1007/s10162-018-0661-0

**Published:** 2018-04-09

**Authors:** Stefan Weder, Xin Zhou, Mehrnaz Shoushtarian, Hamish Innes-Brown, Colette McKay

**Affiliations:** 1 The Bionics Institute, East Melbourne, Australia; 2 Department of ENT, Head and Neck Surgery, Inselspital, Bern University Hospital, Bern, Switzerland; 3 Department of Medical Bionics, The University of Melbourne, Melbourne, Australia

**Keywords:** fNIRS, cortical responses, sound intensity, normal hearing listeners

## Abstract

Sound intensity is a key feature of auditory signals. A profound understanding of cortical processing of this feature is therefore highly desirable. This study investigates whether cortical functional near-infrared spectroscopy (fNIRS) signals reflect sound intensity changes and where on the brain cortex maximal intensity-dependent activations are located. The fNIRS technique is particularly suitable for this kind of hearing study, as it runs silently. Twenty-three normal hearing subjects were included and actively participated in a counterbalanced block design task. Four intensity levels of a modulated noise stimulus with long-term spectrum and modulation characteristics similar to speech were applied, evenly spaced from 15 to 90 dB SPL. Signals from auditory processing cortical fields were derived from a montage of 16 optodes on each side of the head. Results showed that fNIRS responses originating from auditory processing areas are highly dependent on sound intensity level: higher stimulation levels led to higher concentration changes. Caudal and rostral channels showed different waveform morphologies, reflecting specific cortical signal processing of the stimulus. Channels overlying the supramarginal and caudal superior temporal gyrus evoked a phasic response, whereas channels over Broca’s area showed a broad tonic pattern. This data set can serve as a foundation for future auditory fNIRS research to develop the technique as a hearing assessment tool in the normal hearing and hearing-impaired populations.

## Introduction

Sound intensity is a key feature of auditory signals and conveys crucial information about the strength and distance of a sound source. Furthermore, it plays a basic role in many clinical audiology tasks particularly the determination of hearing thresholds and discomfort levels. A profound understanding of cortical processing of sound intensity is therefore highly desirable and might open new possibilities in diagnostics and research. This study investigated whether functional near-infrared spectroscopy (fNIRS) is capable of measuring differences in cortical activation that are related to sound intensity levels and where the maximal level-dependent changes are located on the brain cortex. In this paper, we establish the way that stimulus level affects fNIRS responses in a normal hearing population as a first step to evaluating fNIRS as a tool for hearing assessment in clinical hearing-impaired populations.

Insights about cortical processing of sound intensity have been mainly dominated by animal research. However, animal studies have limitations for assessing perceptual attributes and cannot be directly applied to human conditions. Functional brain imaging methods (such as functional magnetic resonance imaging (fMRI) or fNIRS) enable human in vivo measurements and allow the relationship between physical stimulus properties, stimulus representation in the brain, and their association with perceptual attributes to be studied (Schreiner and Malone 2015).

In the last decade, several groups have used fMRI to study the effect of sound intensity on brain responses. This imaging method allows spatially accurate functional images for all parts of the central nervous system from the brainstem to cortex to be obtained. So far, intensity effects of various stimuli have been studied, including tones (Woods et al. 2009), noise (Sigalovsky and Melcher 2006), dynamic sounds (Seifritz et al. 2002), and speech stimuli (Mohr et al. 1999). It has been shown that the intensity of even simple acoustic stimuli alters the patterns of fMRI activation (Lasota et al. 2003). In the above-cited studies, sound intensities at different levels were applied, from 0 to 100 dB SPL. Röhl and Uppenkamp (2012) found in some participants significant cortical activation down to a level of 20 dB SPL.

Most fMRI research has shown a positive correlation between sound intensity and blood oxygen level-dependent (BOLD) contrast, reflecting increased cortical activation with increased stimulus level (Brechmann et al. 2002; Hall et al. 2001; Hart et al. 2003; Langers et al. 2007; Mohr et al. 1999; Sigalovsky and Melcher 2006). Additionally, the volume of the activated area has been shown to be positively correlated with the stimulus level (Brechmann et al. 2002; Röhl and Uppenkamp 2012). With regard to anatomical locations, most studies reported level-dependent effects in the medial and lateral regions of the superior temporal gyrus including the primary auditory cortex (Hall et al. 2001; Hart et al. 2003; Röhl and Uppenkamp 2012). However, using the fMRI technique in hearing research has restrictions and drawbacks. Besides the often-cited disadvantages such as limited temporal resolution and susceptibility to movement artifacts (Huppert et al. 2006), it can also be more difficult to control stimulus presentation in an MRI acquisition room whereas fNIRS can be easily installed in a sound-treated booth.

With the development of fNIRS, a neuroimaging method has become available that uses near-infrared light generated by LEDs or lasers to determine oxygen saturation changes of the blood. In contrast to fMRI, the technique runs silently allowing more flexible experimental setups in hearing research. Furthermore, it enables measurements in certain population groups, in whom fMRI examinations are not possible or challenging. These include cochlear implant users or newborns and young infants (Olds et al. 2016; Sevy et al. 2010).

The primary aim of this study was to show that different intensity levels of a modulated noise stimulus are related to differences in the fNIRS response. The secondary aim was to delineate brain regions where the fNIRS response shows the highest association with acoustic intensity levels. This study generated data in a normal hearing population, which will serve as a foundation upon which future auditory fNIRS research can be carried out in clinical populations. If fNIRS responses are intensity-dependent, the technique could be useful in clinical populations, where direct verbal feedback is not possible or complicated (e.g., babies and infants). It could help in evaluating cortical activation in response to a certain intensity level and might even assist with adjusting hearing devices (e.g., cochlear implants) in the above-mentioned subpopulation. In contrast to fMRI, fNIRS does not interfere with the cochlear implant coil magnet and is not affected by electrical artifacts as seen in cortical auditory evoked potentials (Sharma et al. 2004).

## Methods

### Study Participants

The study was conducted in accordance with the Declaration of Helsinki, 1975, and had been approved by the local ethical committee (Human Research Ethics Committee, Royal Victorian Eye and Ear Hospital, project number 16/126H). Written consent was given by all participants.

Twenty-four normal hearing, right-handed adults (13 males, 11 females) participated in the study, of which data from 23 were included. Data from one female subject with long dark hair was excluded due to very poor fNIRS signal quality. All participants were healthy with no record of neurological or hearing disorders. No participant used medications that would interfere with cortical activity (e.g., tranquilizers). On the day of the experiment, otoscopy and pure tone audiometry showed normal findings in all participants (hearing threshold less than 20 dB HL at frequencies 125 to 8000 Hz). Furthermore, hearing thresholds for ICRA noise (International Collegium of Rehabilitative Audiology, (Dreschler et al. 2001)) were obtained using a three alternative forced-choice method (Amitay et al. 2006).

### Acoustic Stimulation During the fNIRS Experiment

Auditory stimuli were delivered binaurally via audiometric insert earphones (ER-3A insert earphone, E-A-RTONE™ 165 GOLD, USA). Stimuli consisted of 18-s chunks of the ICRA noise. ICRA noise is completely unintelligible, but has long-term spectrum and modulation characteristics similar to speech (Dreschler et al. 2001). Originally, the ICRA noise was developed for the hearing aid industry to assess technical performance of hearing aids with dynamic compression and noise canceling. Our reason for using a modulated noise stimulus, rather than a simple static stimulus, was to strongly activate broad cortical auditory areas. Firstly, by selecting a broadband stimulus, we aimed to activate a broad cortical region. The bandwidth of auditory stimuli has been positively correlated with the mean percent signal change and spread of cortical activation (Hall et al. 2001). Secondly, ICRA noise is an amplitude-modulated stimulus. fMRI findings have shown that more complex auditory stimuli elicit greater responses in most parts of the auditory cortex (Belin et al. 2002). Thirdly, by choosing a fluctuating stimulus, we aimed to reduce habituation that is seen in multiple repetitions of uniform stimuli (Rankin et al. 2009). And finally, ICRA noise is a well-known and accessible stimulus, allowing other research institutions to reproduce our study design. Despite its amplitude modulation, a precise calibration can be achieved by using the 1-kHz calibration tone included in the stimulus CD (Dreschler et al. 2001).

To account for the variability of this highly modulated stimulus, we used five different chunks of the ICRA noise, randomly assigned to all stimuli used during the fNIRS task. Calibrations were performed using a Norsonic sound level meter (Norsonic SA, Norway) in conjunction with an artificial ear (G.R.A.S., Denmark) according to the ICRA noise compact disc documentation (Dreschler et al. 2001). At the beginning and end of every chunk, a linear on-set and off-set ramp of 10 ms was applied.

### fNIRS Hardware and Experimental Montage

**Fig. 1:**
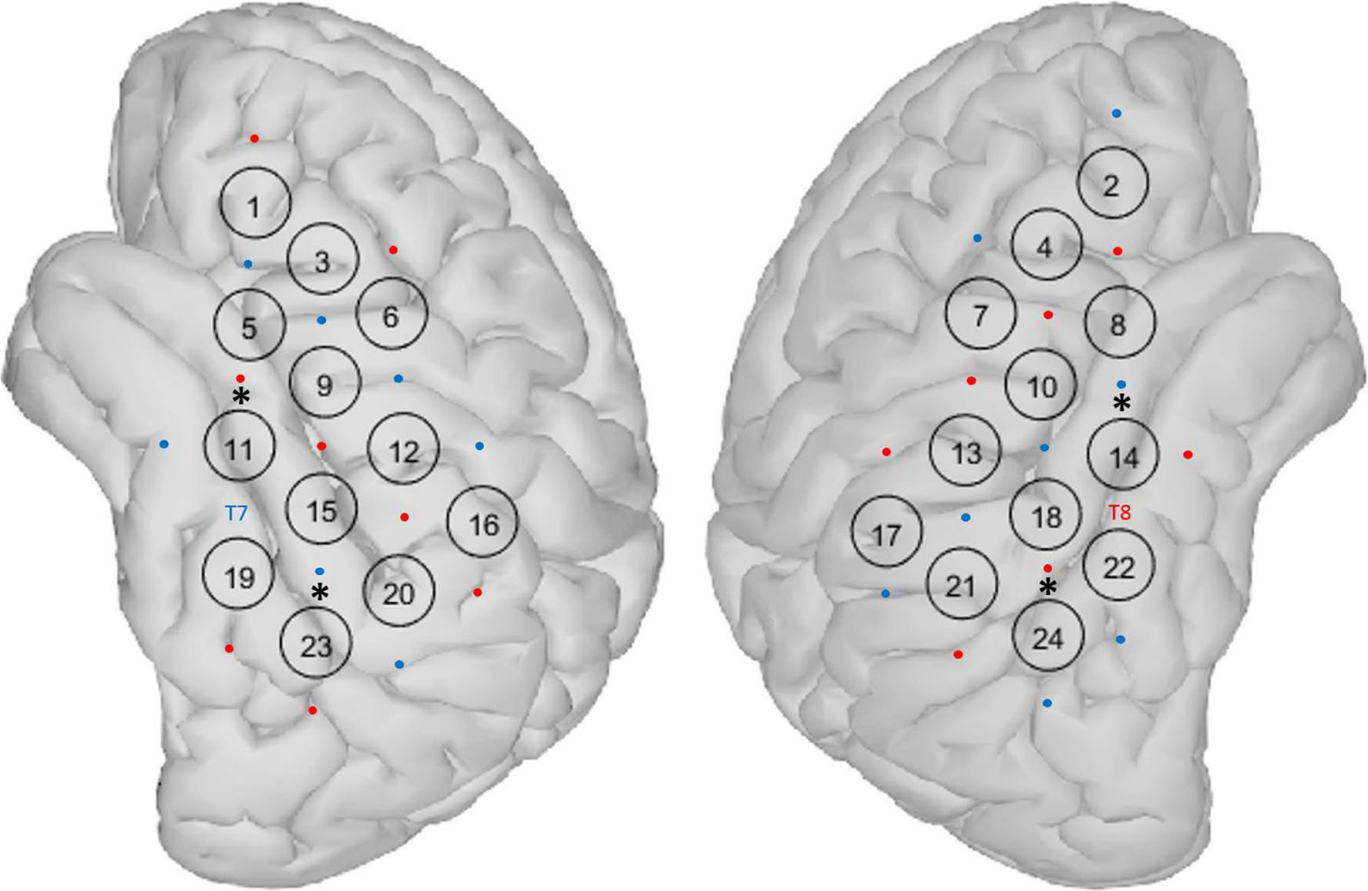
fNIRS montage: eight sources and eight detectors were placed on each side of the scalp (red circles = sources, blue circles = detectors), resulting in16 long channels per side. Above and near the primary auditory cortex channels were overlapping. Two short channels on each side (*) were used for short channel measurements

We used a continuous-wave fNIRS device with a total of 16 LED light sources and 16 avalanche photo-diode light detectors (NIRScout, NIRX, Germany). Sources emitted light of two different wavelengths: 760 and 850 nm. When setting up the cap, the source between channels 11, 15, and 19 and the detector between channels 14, 18, and 22 were placed above EEG positions T7 and T8, respectively (Fig. 1 (Klem et al. 1999)). The cap was then adjusted so that the extension lines between channels 15 and 16 on the left side and channels 18 and 19 on the right side intersected at point Cz on the vertex. The montage of sources and detectors was arranged to create channels (source-detector pairs) with long (3 cm) and short (11 mm) distance (Fig. 1). Above or near the primary auditory cortex, channels were overlapping to generate a more robust signal in this area (channels 11–15, 18, 20, and 21). In long channels, a reliable channel distance of 3 cm was obtained by inserted stabilizing links (NIRX, Germany). Two short-distance channels (11 mm) were included on each side to measure signals arising from the scalp (the asterisk in Fig. 1). These signals were later used to reduce the extracerebral component in long channels as suggested by Sato et al. (2016). In total, 32 long channels were available (16 on each side).

Brain surface MNI coordinates of channel midpoints were determined according to Tsuzuki and Dan (2014). These coordinates were fed into the SPM Anatomy toolbox to allocate them to brain areas according to the Jülich Brain Atlas (Table 1; (Eickhoff et al. 2005)).

**Table 1:** Channel locations channel numbers and the underlying anatomical area for left and right side, respectively, are displayed

Left side		Right side	
CH №	Cytoarchitectonic area	CH №	Cytoarchitectonic area
1	IFG, pars orbitalis	2	IFG, pars orbitalis
3	IFG, pars triangularis	4	IFG, pars triangularis
6	IFG, pars triangularis	7	IFG, pars triangularis
5	Temporal pole	8	Temporal pole
9	IFG, pars operculatris	10	Rolandic operculum
11	Middle temporal gyrus	14	Middle temporal gyrus
12	Postcentral gyrus	13	Postcentral gyrus
15	Superior temporal gyrus	18	Superior temporal gyrus
19	Middle temporal gyrus	22	Middle temporal gyrus
16	Supramarginal gyrus	17	Supramarginal gyrus
20	Supramarginal gyrus	21	Supramarginal gyrus
23	Superior temporal gyrus	24	Superior temporal gyrus

*CH №* channel number, *IFG* inferior frontal gyrus

### fNIRS Testing Procedure and Data Collection

**Fig. 2:**
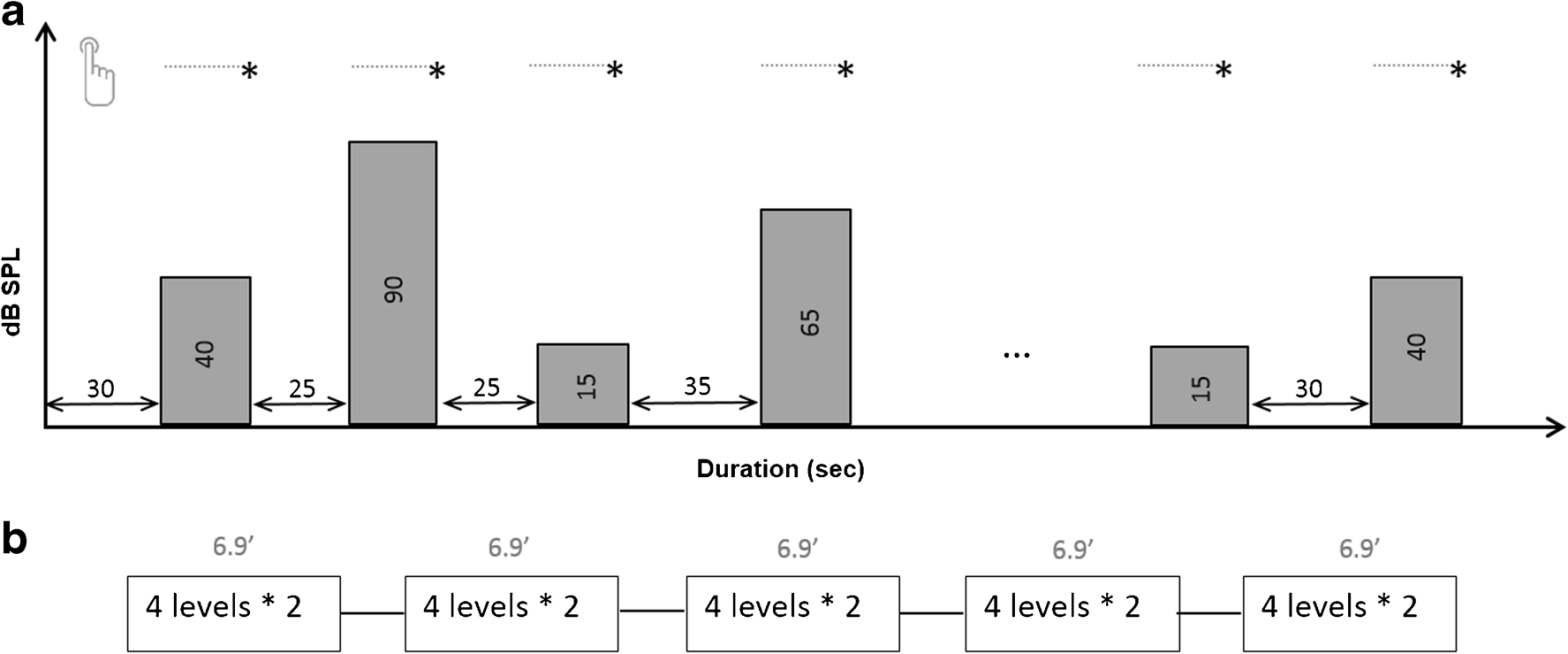
**a** Block design of the fNIRS experiment: four different intensity levels, i.e., 15, 40, 65 and 90 dB SPL, were presented in a counterbalanced order. Blocks were interleaved with resting periods between 25 and 35 s. Participants had to signal the end of a stimulus by a button press. **b** Each testing period (eight stimuli, approx. 7 min) lasted for approximately 7 min. Between testing periods, subjects were given a break of their own chosen duration. In total, each intensity level was repeated 10 times

fNIRS testing was performed in a darkened sound-treated booth. With exception of the fNIRS acquisition equipment, computer hardware was stored outside the testing booth with connection cables running through an insulated hatch to keep the level of background noise as low as possible. The ambient noise was measured at 19.5 dBA. Participants were sitting in an armchair and were asked to fixate on a white cross on a monitor during recording sessions. For comfort and to reduce movement artifacts, the participant’s neck was stabilized using a neck cushion. Stimulus presentation and trigger recording were performed using the Presentation software (Neurobehavioral Systems, USA). ICRA noise stimuli of different intensity levels (15, 40, 65, and 90 dB SPL) were presented in a counterbalanced block design starting with a baseline of 30 s resting period (Fig. 2). The 15-dB SPL stimuli were just above hearing threshold for the participants, as confirmed by the threshold test using ICRA noise.

The fNIRS test session consisted of five test periods of 7 min each (35 min total recording time). In each of the five test periods, each intensity level was presented twice in a counterbalanced order. In total, each intensity level was repeated 10 times. Each auditory stimulus was followed by a rest period (silence) with randomly chosen duration of 25, 30, or 35 s. Between test periods, participants took a break of their own chosen duration. The fNIRS cap remained on the participant’s head during the whole test session, including the breaks.

Participants were instructed to pay attention to any incoming sound. To maintain their attention to the stimuli and to evaluate stimulus recognition later on, they were asked to push a button at the end of each stimulus (Fig. 2). After completing fNIRS recordings, subjects were asked how well they had focused on the task (“not at all,” “mostly not,” “sometimes,” “mostly always,” “always”) and were asked to estimate their level of mental exertion during the experiment on a modified Borg scale (Borg 1982), ranging from 0 (“no exertion at all”) to 10 (“maximal exertion”). This questionnaire was applied in order to assess whether alteration of attention (Woods et al. 2009) or increased task demands (Church et al. 2010) were likely to modulate the neural activation to the stimuli.

### Data Pre-processing and Analysis

Data pre-processing and analysis was executed in MATLAB (MathWorks, USA) and SPSS (version 24, IBM Corp., USA). Custom-made MATLAB scripts were combined with Homer2 functions (Huppert et al. 2009). The following steps were executed:

A. Channels with unusable data were excluded from further analysis. Channels with insufficient optode contact to the scalp (indicated by a system gain higher than a limit) were rejected, as were channels in which the scalp coupling index (SCI) was less than 0.75 (Pollonini et al. 2014). The SCI (between 0 and 1) signifies the cross-correlation between the two wavelengths essentially due to strong cardiac signals in the raw data. A high SCI (> 0.75) is an indication that both optodes that made up the channel were well coupled to the scalp.
B. The hemodynamic response was extracted using the following steps. Raw data were converted into optical densities. Motion artifacts were removed by using wavelet transformation of the data (Molavi and Dumont 2012). The Homer2 toolbox bandpass filter (0.01 and 0.5 Hz; (Huppert et al. 2009)) reduced drift, broadband noise, heartbeat, and respiration artifacts. Concentration changes of oxygenated (HbO) and deoxygenated hemoglobin (HbR) were estimated by applying the modified Beer-Lambert law (Delpy et al. 1988). Lastly, the extracerebral component in long channels was reduced by using measurements from the short channels as follows: the first principal components (PC1) from the two short channels on each side were estimated. For each side separately, a general linear model (GLM), consisting of the hemodynamic response functions of the stimuli (Kamran et al. 2015) and the PC1, was used to fit the signals in long channels. Finally, the extracerebral response, i.e., PC1 multiplied by its coefficient from the GLM, was subtracted from the signal in each long channel as described by Sato et al. (2016).

For all channels separately, block-averages of the signals from step B were computed by taking median values across all 10 epochs for a particular stimulus. Before computing the median value, studentized residual analysis was used to detect outliers in each epoch, as described by Huppert et al. (2009). Epochs, where residuals exceeded 2.5 standard deviations above the mean during baseline and very early stimulation phase [− 5 to + 2 s], were discarded. Responses in overlapping channels (Fig. 1b) were averaged, resulting in a total of 12 channels on each side.

The waveform morphology was explored on a channel-by-channel basis. However, for statistical analyses, our whole montage was divided into regions of interest (ROIs). ROI data is advantageous in statistical comparisons, as it allows single channels to be excluded if necessary, it limits the need for multiple statistical comparisons, and it gives a more simplified overview for the reader. Three neighboring channels were combined to form each ROI and were selected according to similar grand average waveform patterns present in both oxygenation states (Fig. 5). As our main goal was to evaluate the effect of level on the neural response and not to evaluate or interpret spatial patterns with anatomical structure or functions, we chose this method of selecting ROIs as the method that most supported our aim. As our data were not normally distributed, non-parametric tests were utilized for statistical testing. To account for multiple comparisons, Bonferroni correction was applied if indicated.

## Results

### Behavioral Data

**Fig. 3:**
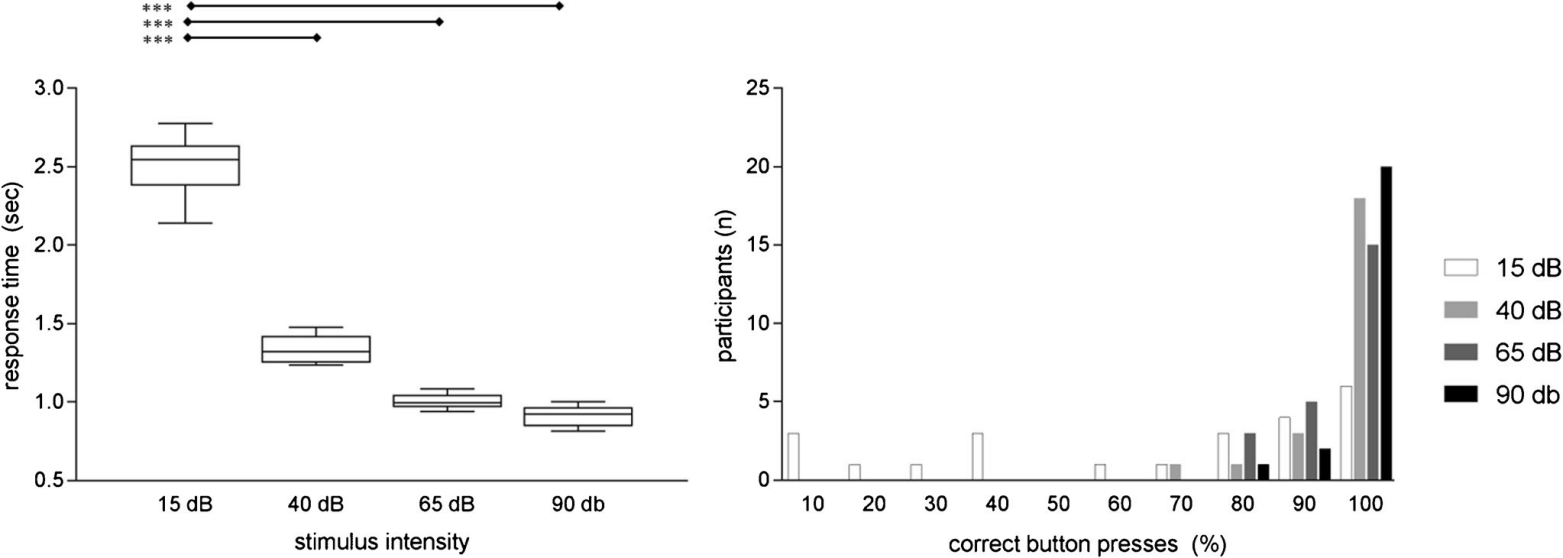
**a** Time to button press in seconds. **b** Percentage of correct button presses for different intensity levels. For the lowest level (15 dB), participants pressed the button significantly later (***p < 0.001) and the percentage of recognized stimuli was much lower than for the higher-level stimuli

For all participants, hearing thresholds for ICRA noise were closely clustered (average 10.4 dB SPL, range 6–14.5 dB SPL). Each participant’s threshold for the ICRA noise was therefore close to the lowest presentation level (15 dB SPL). This finding was additionally supported by button press data. Whereas most other stimuli were promptly indicated with a button press, at 15 dB level, many stimuli were missed, or participants were unsure when the stimulus had ended as reflected in a delayed button press (Fig. 3).

Most participants reported that they were focused on the task (1 “sometimes,” 15 “mostly always,” and 7 “always”). Responses from the modified Borg scale (0–10, see the “fNIRS Hardware and Experimental Montage” section) indicated that participants found the task to be undemanding. The median of estimated mental exertion was 2 (“slight mental exertion”) with most indications between 1 and 4. Only one participant stated a high number (9), as he was feeling very sleepy in the darkened sound booth and struggling against falling asleep. Therefore, we assumed that participants were on average focused on the task and our task demand did not influence fNIRS responses significantly.

### fNIRS Grand Average Waveform Morphology

**Fig. 4:**
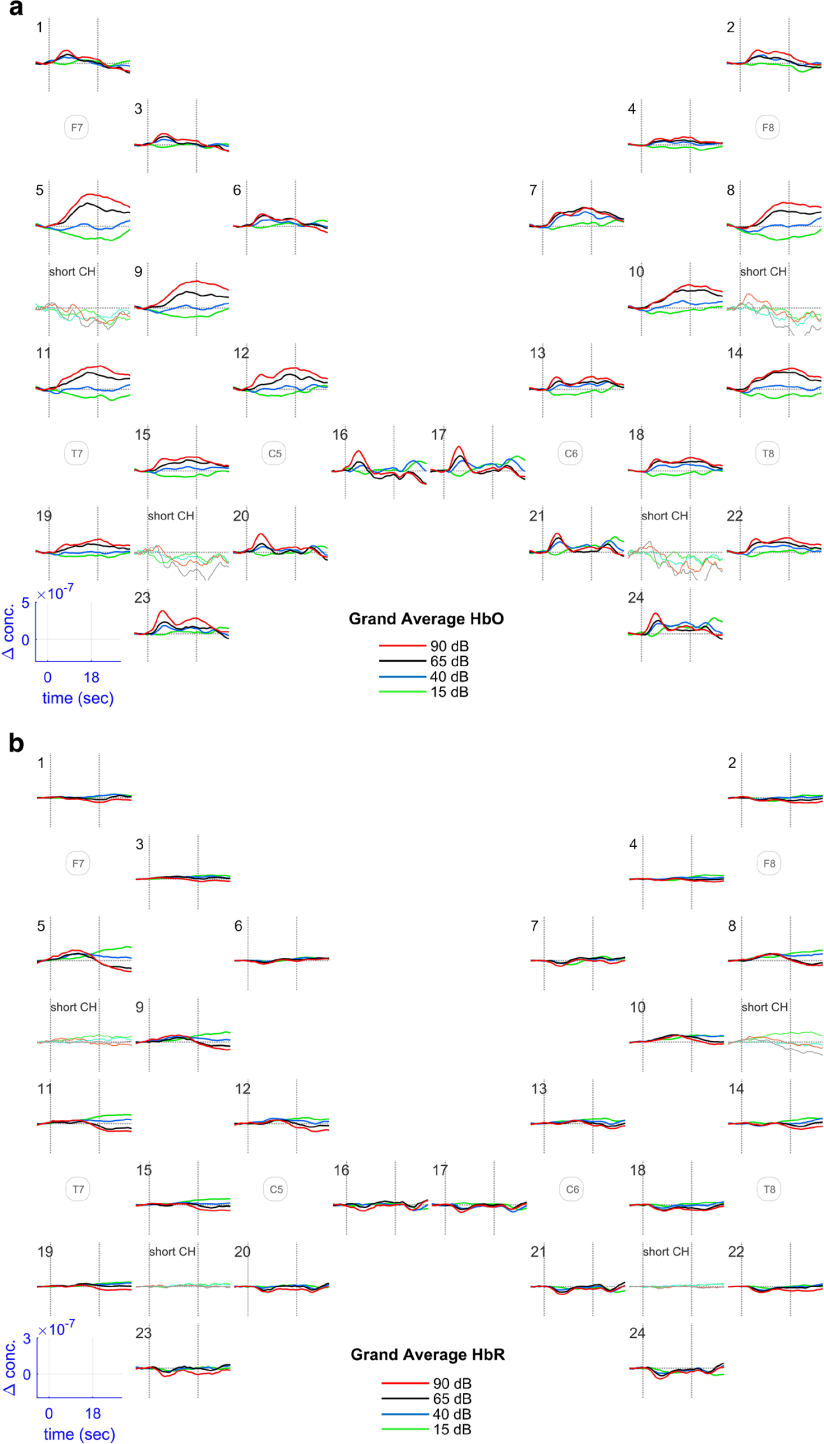
Grand average channel data for HbO (**a**) and HbR (**b**) concentration changes. Channel numbers are delineated in the upper left corner of every plot. For additional spatial orientation, 10–20 positions are included in the plot. Responses to different intensity levels are color encoded. The blue box in the lower left corner delineates the scales of the *x*- and *y*-axis; dotted vertical lines correspond to the stimulus on- and off-set. Additionally, the grand averages of the two short channels are displayed on each side

Grand average concentration change data for HbO (Fig. 4a) and HbR (Fig. 4b) were generated. Two main waveform patterns were present—a phasic response and a tonic response—both symmetric across the left and right hemisphere.

Phasic responses were found in channels that were the most caudal (channels 16, 20, and 23 on the left and channels 17, 21, and 24 on the right side) and the most rostral (channels 1, 3, and 6 on the right and channels 2, 4, and 7 on the left side). These channels showed a sharp peak in the first half of the stimulation period. Rostral channels only showed an on-set peak, whereas caudal channels also exhibited an off-set peak occurring a few seconds after stimulation had ended. Tonic responses were found in channels 5, 9, and 11 on the left and channels 8, 10, and 14 on the right side. No on- or off-set peaks were visible in these channels, but rather a steady and slow increase in HbO concentration during the stimulus period reaching a peak before the stimulus off-set and a slowly declining thereafter. For the lowest intensity level (15 dB SPL) a negative concentration change was noted. Finally, channels 12, 15, and 19 on the left side and channels 13, 18, and 22 on the right side showed a mixed pattern with a peak in the first half of the stimulation period, transitioning into the broad pattern of channels 5, 9, and 11 and 8, 10, and 14, respectively.

Time-lagged HbR responses evolved reciprocally to HbO (Fig. 4). The time lag of HbR compared to HbO varied from back to front regions: for caudal channels, HbR nadirs appeared approximately 2 s after HbO peaks. For channels with the phasic response pattern, an initial small increase in HbR was seen, followed by a progressive decrease with its minimum after stimulus off-set.

### fNIRS Signal Change in Response to Increasing Intensity Levels

**Fig. 5:**
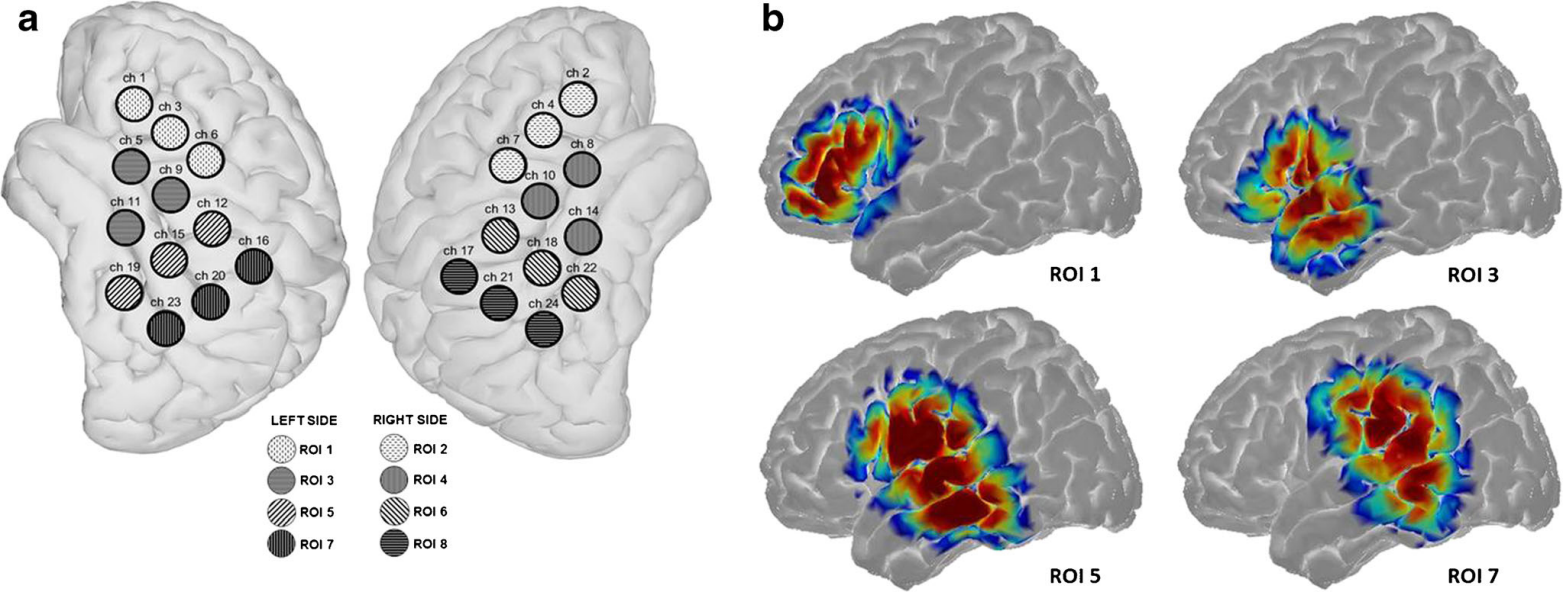
**a** Based on the pattern of grand average data, four regions of interest were created for the left and right hemisphere. **b** Sensitivity maps using Montecarlo simulation display the cortical areas to which the different ROIs are sensitive (here only shown for the left side)

For statistical testing, and due to the distinct waveform pattern that was symmetric across hemispheres, ROIs were created (Fig. 5). For each ROI, three neighboring channels with similar waveform patterns in HbO and HbR were averaged (Fig. 5a). As diffuse optical imaging has limited spatial resolution, Homer2’s inbuilt Monte Carlo simulation was run to obtain a sensitivity measure of the brain regions covered by the selected ROI’s (Fig. 5b, (Aasted et al. 2015)).

**Fig. 6:**
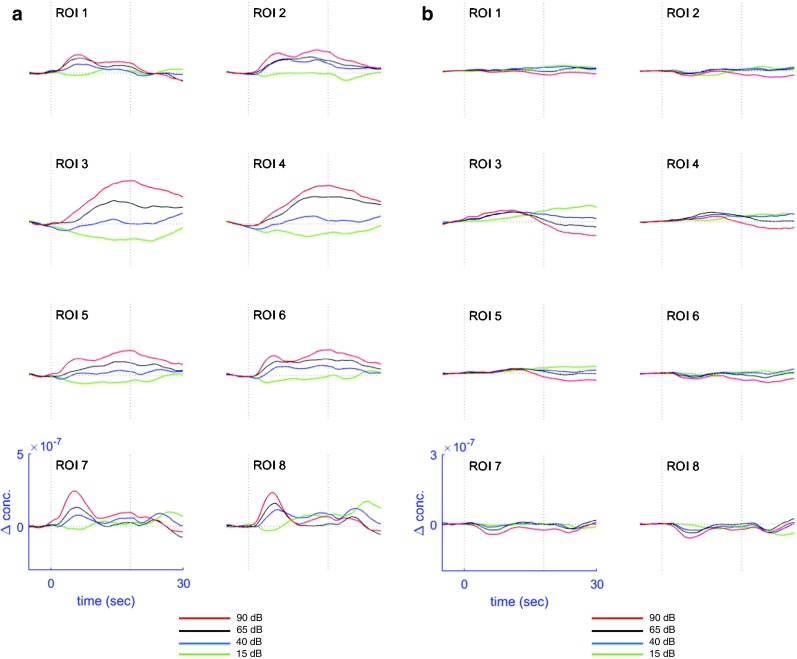
fNIRS responses for ROIs in relation to different sound intensity levels, for HbO (**a**) and HbR (**b**), respectively. The blue box in the lower left corner delineates the scales of the *x*- and *y*-axis. The horizontal dashed line underneath each figure displays midpoints of moving windows (± 3 s, *p* ≤ 0.05) with significant effect of intensity level as calculated by Friedman’s test. The red cross points out the midpoint of the window with the highest chi-square statistics

Figure 6 shows the time-course of HbO and HbR ROI data (Fig. 6a, b). In all regions, higher simulation levels led to an increase of HbO and a time-lagged decrease of HbR. However, morphologically, the time point of maximal concentration changes differed according the ROI. Caudal ROIs 7 and 8 showed the biggest difference in grand average response amplitude for different intensity levels during the on-set peak, whereas more rostral ROIs 3 and 4 reached the point of maximum difference between intensities much later in the second half of the stimulation period. Interestingly, the off-set peak of regions 7 and 8 showed an inversed pattern of the waveform order: highest intensity levels led to the smallest concentration change and vice versa.

**Fig. 7:**
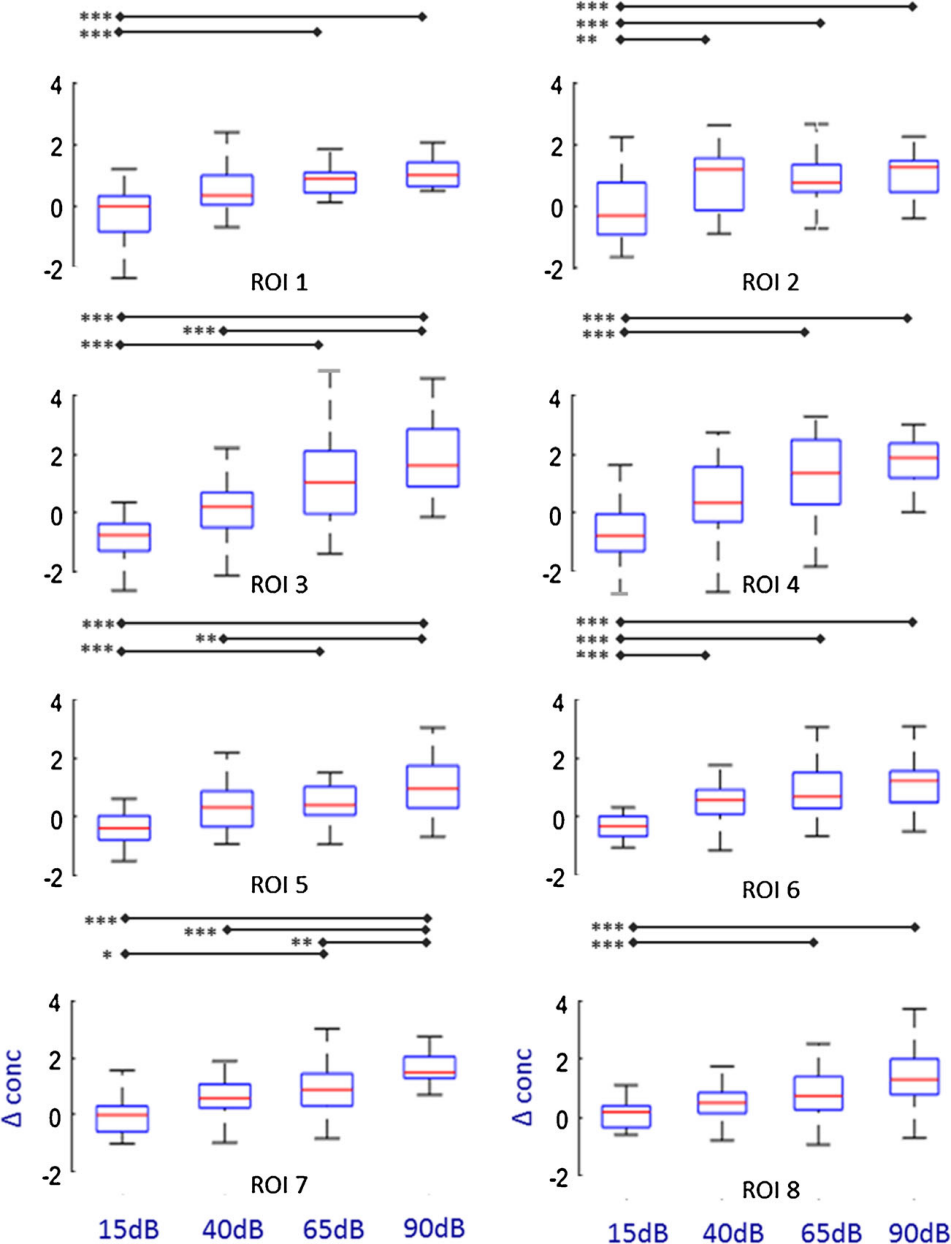
Boxplots showing the distribution of responses for every ROI and intensity level for the 6-s time window with the highest chi-square statistics. The red lines show the median responses (*y*-axis: delta concentration change in arbitrary units *10^′−7^). Above every panel, statistically significant post hoc comparisons are shown (*p < 0.05, **p < 0.01, ***p < 0.001)

To explore the time intervals within which a significant effect of stimulus intensity was present, we used a 6-s moving window. In each window, the average response for each subject for each intensity level and oxygenation state was calculated. Then, the effect of intensity level was evaluated using Friedman’s test. Figure 6 displays midpoints of significant moving windows (*p* ≤ 0.05) for each region separately (dotted line). In Table 2, the time window with the highest chi square statistics is displayed for every ROI and oxygenation state, separately. For these particular time windows, post hoc comparisons were executed using Wilcoxon signed-rank tests (Fig. 7).

**Table 2:** Statistical analysis of effect of intensity: Friedman’s test for every region of interest (ROI, A) and for representative channels (CHL, B) separately, “*t*-window” displays the midpoint of the time window with the highest chi square statistics (*X*^2^, in () degrees of freedom) and its corresponding Bonferroni corrected *p* value

		A) HbO			B) HbR	
A)						
ROI	*t*-window	*X*^2^ (3)	*p* value	*t*-window	*X*^2^ (3)	*p* value
1	8	24.7	< 0.000	20	18.1	0.002
2	8	22.5	< 0.000	15	4.1	0.251
3	18	30.7	< 0.000	24	23.0	< 0.000
4	14	28.0	< 0.000	27	9.4	0.049
5	9	35.4	< 0.000	25	19.5	0.001
6	8	37.7	< 0.000	8	11.4	0.029
7	6	43.5	< 0.000	6	19.1	0.001
8	5	26.6	< 0.000	7	22.5	< 0.000
B)						
ChL	*t*-window	*X*^2^ (3)	*p* value	*t*-window	*X*^2^ (3)	*p* value
5	11	29	< 0.000	27	18	0.002
8	14	28	< 0.000	25	17.8	0.251
20	6	31	< 0.000	7	13.5	< 0.000
21	7	23	< 0.000	6	19.7	0.049

For HbO responses, changes in sound intensity level led to highly significant changes in all eight regions (Table 2, Figs. 6 and 7). Moreover, statistical testing confirmed morphological findings: the significant time windows where the response amplitude for different intensities differed significantly varied according to the ROI. ROIs 7 and 8 reached their most significant time window (highest chi square statistics) early after stimulus on-set (when the midpoint of the moving window was 5 and 6 s after stimulus on-set). In contrast, ROIs 3 and 4 showed a much slower pattern with highest chi square statistics at seconds 18 and 14 (midpoint of moving window). Finally, ROIs 1, 2, 5, and 6 showed an early temporal pattern with the most significant time windows in the first half of the stimulation period (midpoints between 8 and 9 s). Of all regions, ROI 7 showed the highest significance level and greatest number of significant pairwise comparisons between response amplitudes at different intensities. On the other hand, ROIs 3 and 4 displayed the largest average responses to the stimuli but with a higher variance among subjects than ROIs 7 and 8 (Fig. 7).

For HbR, all regions except ROI 2 showed a significant effect of stimulus intensity (Table 2). Rostral regions (ROIs 1 to 5) showed the highest chi-square statistics in late time windows, whereas back regions (ROIs 6 to 8) exhibited the most significant effect in early time windows.

For the reasons given above, ROI instead of channel data was compiled for statistical analysis. However, to determine whether our results may have been due to particular features of the ROIs used, we repeated the analysis for two representative channels on each side (channels 5 and 20 on the left and channels 8 and 21 on the right side representing the two morphological patterns). The results were consistent with the ROI data (Table 2).

### Laterality of Brain Activation

To test for side preferences of brain activation, a Mann-Whitney *U* test was run. HbO responses were integrated over the following time periods after stimulus on-set: 0–10, 10–20, and 20–30 s and compared between corresponding ROI’s in each hemisphere (e.g., ROIs 1 and 2). On a ROI basis, the integrals of HbO and HbR responses were similarly distributed for left and right side respectively (*p* > 0.05). These findings were verified on a channel-by-channel basis, with no significant differences between hemispheres.

## Discussion

In this study, we investigated the effect of sound intensity on fNIRS signals originating from the temporal cortex and propagating over multiple cortical areas. The results revealed a significant effect of intensity level on cortical hemodynamics as expressed by hemoglobin saturation change. For caudal and rostral regions, different waveform morphologies were noted, reflecting specific cortical signal processing of the modulated noise stimulus.

We assume that the processed responses reflect functional brain activity, as the task was well defined and easily feasible, we reduced extracerebral signals by short-channel PCA correction, and HbO and HbR were anti-correlated—a characteristic sign for the biological validity of fNIRS data (Tachtsidis and Scholkmann 2016).

### Effect of Sound Intensity

Our data demonstrates a clear and significant effect of sound intensity on HbO and HbR concentration changes. This effect was present in caudal areas, with on- and off-set response peaks, and in rostral regions, with broad and sustained responses. Our results are consistent with findings from previous fMRI and PET-CT studies: i.e., higher intensity levels lead to an increased activation of auditory cortical fields (Langers et al. 2007; Lockwood et al. 1999; Sigalovsky and Melcher 2006). fNIRS studies investigating the effects of stimulus intensity are sparse and have been contradictory so far. In a combined fNIRS-EEG study, Chen et al. (2015) found no statistically significant effect of higher intensity levels on hemoglobin saturation changes. However, in a subgroup, they found a correlation between loudness and HbR (Chen et al. 2015). In a case series from 2016, a significant difference in fNIRS responses between two intensity levels was found in three out of four participants (Bauernfeind et al. 2016). In the latter study, a subtraction map (loud vs. quiet stimulus) showed a broad activation pattern in channels above Broca’s area, whereas more caudally positioned channels (i.e., above supramarginal gyrus) displayed a reduction in activity for high compared to low intensities.

In our HbO data, a significant effect of sound intensity was noted in all regions. However, the most significant chi-square statistic was found in the most rostral region with a phasic waveform morphology (ROI 7). This brain region has been shown to be particularly activated in phonological processing of words (Hartwigsen et al. 2010; McDermott et al. 2003) as well as speech modulated noise (Giraud et al. 2004). However, the latter study showed that this region is similarly activated independently of whether the stimulus was intelligible or not, suggesting that the region is sensitive to complex modulated stimuli (such as the one used in the current study) rather than being an area specific for speech comprehension.

In our data, the largest responses to the stimuli, but with a higher variance among subjects, were observed in the regions with a tonic response (ROIs 3 and 4). fMRI studies have also found increased cortical activation due to higher intensity levels in primary and secondary auditory areas (Jäncke et al. 1998; Langers et al. 2007; Sigalovsky and Melcher 2006). In our data and in accordance with the fMRI literature (Jäncke et al. 1998; Langers et al. 2007), a response saturation for the highest intensity levels was not seen.

In our experiment, we could not detect performance monitoring in rostral channels as described by Dosenbach et al. (2007). In ROIs 1 and 2, higher stimulation levels (which were easily detectable) led to significantly higher cortical activation compared to the lowest stimulation level (which were hardest to detect). Hence, our findings are not likely to reflect task control where the opposite pattern would be expected.

In relation to hemisphere differences, although there were small morphological differences between the left and right hemisphere (e.g., between channels 6 and 7; Fig. 4a), these differences were not statistically significant. The lack of lateralization of responses might be explained by the facts that our stimulus was delivered binaurally and the task demands were restricted solely to the perception of an elementary sensation without any further cognitive processing. A salience effect, which would tend to be more right lateralized, would be unlikely with our study protocol (Mueller-Pfeiffer et al. 2014).

Finally, on-set and sustained responses increased with higher intensity levels, whereas off-set peaks in the most caudal regions showed an inverse pattern. We speculate that this finding could be explained by changes in attention allocated to the stimuli of different levels: as the lowest intensity level was near hearing threshold, participants perceived peak amplitudes only. Correct timing of the button press after stimulus off-set was therefore difficult. Such an inverse response pattern to activation tasks is typical of areas belonging to the default mode network and this effect has been shown to be independent of task modality (Gusnard and Raichle 2001; Raichle 2015). Task-independent decreases in the posterior lateral cortices have been observed in the supramarginal and angular gyrus (Shulman et al. 1997), which may have been of significance in our task when focus turns away from internal mental activity to acoustic information processing.

### Waveform Morphology

Our data show a distinct pattern which was symmetric across the hemispheres and restricted to designated channels. The shape of the responses was highly dependent on the brain region: caudal channels (channel midpoints above supramarginal and superior temporal gyrus) showed on- and off-set peaks (phasic responses), whereas channels with midpoints above anterior superior temporal gyrus and Broca’s area displayed a slowly developing sustained response (tonic response). Waveform morphology also determined the time window with the highest effect of sound intensity. Channels with phasic responses showed the most significant effect of intensity in early time windows, whereas channels with tonic waveforms exhibited the most significant effect of intensity in the second half of the stimulation period.

In fMRI studies, phasic and tonic waveform responses are well-known. These different activation dynamics have to be taken into account when processing and interpreting functional imaging data (Harms and Melcher 2003). Interestingly, one of the two fNIRS intensity studies also reports a tonic on- and off-set peak pattern in their measured signals (Chen et al. 2015). It has been demonstrated that higher sound repetition rates (35/s) lead to a phasic response (with an on- and off-set peak) and slower repetition rates (1/s) to a tonic response in the primary auditory cortex and its neighboring superior temporal gyrus (Harms and Melcher 2002). The high modulation of our stimulus (with main modulation frequencies in the 2 to 20 Hz range) might therefore explain the phasic response for caudal channels, which lie near the primary auditory cortex. Furthermore, Sigalovsky and Melcher (2006) et al. demonstrated in an fMRI experiment that the waveform pattern (i.e., phasic and tonic responses) can change across different stages of the auditory pathway: the same stimulus elicits different patterns in the brainstem and the primary and secondary auditory fields. In our data too, the waveform shape is stimulus but also location dependent: channels above the temporal pole and motor speech area (Broca’s area) elicit tonic responses. In fMRI, speech-envelope noise has been shown to elicit tonic responses in both anterior superior temporal gyri (Giraud et al. 2004). This brain region is highly sensitive to temporal sound envelopes such as those which are present in speech signals (Giraud et al. 2000). The ICRA noise used in our study, although unintelligible, has spectral and temporal characteristics similar to speech.

### Limitations and Outlook

In hearing studies, the most striking advantage of fNIRS is that the technology runs silently. Furthermore, compared to fMRI, it is less susceptible to movement artifacts, has a higher time resolution, and is applicable in children and patients with implanted devices. However, limitations have to be considered. Measurements are restricted to the outer cortex of the brain. It is therefore disputable whether fNIRS can detect signals in adults that originate from the primary auditory cortex, which lies in the depth of the lateral sulcus. It is more likely that optodes over this region pick up signals from the para-belt region instead (Wiggins et al. 2016). Furthermore, the spatial resolution of fNIRS is limited and it must be assumed that channel responses originate not only from channel midpoints but from broader cortical areas (as outlined in the probability map, Fig. 5b). Without simultaneous MRI imaging, the localization of fNIRS signals are only an approximation. Lastly, continuous-wave fNIRS allows two-dimensional measures only; estimations of activated volume changes, as described in fMRI studies, are not possible.

Further research is needed to better understand the morphological response patterns due to stimulus properties. This point is of importance, as many functional imaging studies use predictive models based on a fixed morphology, which might significantly influence the results. For clinical studies, more frequency specific stimuli would be desirable. Further, in fNIRS studies, a consensus concerning optimal study protocols (e.g., stimulus duration, repetition rate) has yet to be reached and needs to be further assessed. Future fNIRS studies investigating the effect of sound intensity must address whether cortical activations can also be measured in hearing-impaired people and populations where attention cannot be controlled (e.g. infants).

## Conclusion

The fNIRS technique is particularly suitable for this kind of hearing study, as it runs silently. Measurements can be performed without interference from hearing devices such as cochlear implants. Our study results showed that fNIRS responses are highly dependent on sound intensity level. Our modulated noise stimulus with long-term spectrum and modulation characteristics similar to speech elicited specific response patterns depending on the overlying brain region. Channels overlying the supramarginal and caudal superior temporal gyrus evoked a phasic response. In this region, the most statistically significant effect of sound intensity can be found in early time windows during stimulation. Channels covering the antero-superior temporal gyrus and Broca’s areas showed a broad tonic pattern, where a significant effect of sound intensity level can be observed in late time windows.

This study was the first step towards developing the fNIRS technique as hearing assessment tool. This data set of a normal hearing population can serve as a foundation for future auditory fNIRS research in clinical populations.
